# Pain Predicts Function One Year Later: A Comparison across Pain Measures in a Rheumatoid Arthritis Sample

**DOI:** 10.1155/2016/7478509

**Published:** 2016-03-28

**Authors:** Vivian Santiago, Karen Raphael, Betty Chewning

**Affiliations:** ^1^New York University College of Dentistry, New York, NY 10010, USA; ^2^University of Wisconsin at Madison, Madison, WI 53705, USA

## Abstract

*Background*. Guidance is limited on best measures and time periods to reference when measuring pain in order to predict future function.* Objective*. To examine how different measures of pain predict functional limitations a year later in a sample of rheumatoid arthritis patients.* Methods*. Logistic regression analyses were conducted using baseline and one-year data (*n* = 262). Pain intensity in the* last 24 hours* was measured on a 0–10 numerical rating scale and in the* last month* using an item from the Arthritis Impact Measurement Scale 2 (AIMS2). AIMS2 also provided frequency of severe pain, pain composite scores, and patient-reported limitations. Physician-rated function was also examined.* Results*. Composite AIMS2 pain scale performed best, predicting every functional outcome with the greatest magnitude, a one-point increase in pain score predicting 21% increased odds of limitations (combined patient and physician report). However, its constituent item—frequency of severe pain in the last month—performed nearly as well (19% increased odds). Pain intensity measures in last month and last 24 hours yielded inconsistent findings.* Conclusion*. Although all measures of pain predicted some functional limitations, predictive consistency varied by measure. Frequency of severe pain in the last month provided a good balance of brevity and predictive power.

## 1. Introduction

Pain is a core symptom of rheumatoid arthritis (RA); therefore, its valid assessment is critical for RA research and clinical management. Although the reliability and validity of common measures of pain intensity have been established [1], studies seldom explore what time period these measures should assess to best guide treatment decisions.

Despite the well documented negative impact of chronic pain on health related quality of life and functional outcomes [2, 3], including significant impact on disability in RA [4–7], how best to measure pain to predict future RA specific functional outcomes has not been adequately studied. Most research on the measurement of pain in RA focuses on the reliability of measures [8] rather than their utility in predicting the course of illness. Studies that do explore the role of pain as a predictor of functional disability focus on concurrent pain [9] or treat pain as one of many variables when examining stronger predictors of future function [10]. Since factors other than pain are the main predictors of interest, these studies fail to fully characterize the association of pain with future function and, more importantly, do not explore how different measures of pain and the time periods they reference may impact results. Therefore, there is little evidence to help guide clinicians and researchers on how best to assess RA pain to focus treatment and/or intervention on patients who are most likely to have impairment in function over time.

In the context of clinical trials for pain in general, IMMPACT recommendations [11] outline* pain intensity* as one key outcome measure, recommending primarily the use of a 0–10-point numerical rating scale for rating pain. Recommendations also suggest use of a verbal rating scale that may be easier for some patients to complete. Recommendations consider the specific anchoring of pain measures to rating the worst or least pain experience and mention use of last 24 hours or last week, depending on the research questions. Although they acknowledge the utility of disease specific measures of pain, additional evidence-based guidance on which time frame may be best to assess for a given research or clinical purpose is not provided and difficult to find.

This study examines how different pain measures at one point in time predict functional limitations a year later in a sample of rheumatoid arthritis patients. Specifically, two time frames for measuring pain intensity at initial study assessment (i.e., the last 24 hours or the last month) are evaluated to determine which better predicts functional outcomes at one year among RA patients receiving routine rheumatologic care. In addition, results are compared to those obtained from a composite measure of RA pain and a single item measure of frequency of severe pain to determine which approach may be most useful in predicting functional limitations one year later.

## 2. Materials and Methods

### 2.1. Sample

Specific aims were examined with the use of data from* The Older Adults and Drug Decisions Study*, a completed multisite study of patients in 6 rheumatology clinics (2 in Wisconsin and 4 in North Carolina), funded by the National Institutes on Aging [12, 13]. The study followed patients over 1 year with an 86% participation rate and 78% retention rate. At the 6-month visit, patients were asked via computer assisted interviews to either rate health priorities (the intervention) or complete a lifestyle questionnaire (the control group) prior to meeting with the clinician. Patients were then followed up for another 6 months.

Subjects were enrolled in the parent study if they were patients of participating rheumatologists, were at least 45 years old, were diagnosed with RA, and were able to communicate in English. Patients who had 5 or more errors on the 10-point Mental Status Questionnaire [14] were not eligible for participation. The current analyses use observations with complete data on the relevant measures of pain and function at baseline and function at the 1-year assessment (*N* = 262). The original intervention of the parent study is not of interest in these aims but is explored as a covariate. Sample characteristics are summarized in Table 1.

### 2.2. Measures

All patient-reported measures were collected via research interview or self-reported survey after each medical visit. Physician ratings of function were abstracted from the medical records.

#### 2.2.1. Pain Measures and Referent Time Frame

Pain intensity was measured via a numerical rating scale and the pain scale of the Arthritis Impact Measurement Scale 2 (AIMS2) [8]. First, to measure pain intensity in the last 24 hours, the numerical rating scale was used. This measure included a numbered line and asks patients to rate pain intensity from 0 to 10 with 0 as* no pain* and 10 as* worst pain ever*. Second, for pain intensity during the last month, the first question of the AIMS2 pain scale was used which asks patients,* during the past month how would you describe the arthritis pain you usually had?* Response options included* severe, moderate, mild, very mild, *and* none*. For comparison with other measures, responses were converted to a 0–10 scale, 10 representing severe pain, as is done when scoring the AIMS2 scales. Third, frequency of severe pain was also measured with a single item from the AIMS2 pain scale which asks:* during the past month, how often did you have severe pain from your arthritis?* Responses included* all days, most days, some days, few days, *and* no days*, also converted to a 0–10 scale, 10 representing all days for analysis.

Fourth, the AIMS2 full pain scale was used as a composite measure of pain, which includes 5 items. In addition to the above noted measure of pain intensity and frequency of severe pain in the last month, the composite score also includes an item on frequency of* pain in 2 or more joints*, frequency of* morning stiffness lasting longer than 1 hour after waking*, and frequency of* pain making it difficult to sleep*. The scale for these items was the same as for frequency of severe pain:* all days, most days, some days, few days, *and* no days*, 10 representing all days and 0 no days. These 5 items were averaged to yield a composite score on a 0–10 scale per AIMS2 scoring guidelines [8], with 10 indicating most frequent/severe pain.

Each of these measures has been found to have good psychometric properties including good reliability and validity [1, 8, 15].

#### 2.2.2. Functional Limitations

Functional limitations were measured via self-report using the mobility and self-care subscales of the AIMS2. The AIMS2 is an arthritis-specific self-reported measure of function that has been found to have good psychometric properties [8] and correlate with general measures of health and function like the SF-36 and the Modified Health Assessment Questionnaire [16].

Functional outcomes had highly skewed distributions with sparse cells and many observations with no functional limitations; therefore, they were dichotomized (Table 1). Self-reported outcomes on mobility and self-care limitations were dichotomized to represent a score of at least 1 out of 10 reported versus less than 1 on the original scales (<1 versus ≥1).

In addition to the patient-reported outcomes, during the physical exam, clinically assessed function was rated by physicians using the American College of Rheumatology (ACR) classification of global functional status in rheumatoid arthritis, which includes 4 classes [17]. ACR functional status was dichotomized in similar fashion, class I representing no limitations versus classes II through IV representing any limitation. To combine patient and physician reports, one combined dichotomous measure of function was calculated as limitations if at least 1 of the patient reported dichotomous measures of function was positive* and* the ACR physician reported measure was positive for functional limitations.

#### 2.2.3. Sociodemographic and Other Variables

Specific sociodemographic variables explored included age, gender, educational attainment, marital status, race, state of study site, and exposure or control status, all measured at baseline. Only variables associated with any functional outcome and pain measures were considered for inclusion. Variables included marital status defined as married versus other including single or widowed, education dichotomized as greater than high school versus high school/less than high school, geographic state (Wisconsin or North Carolina), or race defined as white versus all other races. As a proxy for patient's overall health at baseline, self-rated health was measured using an item from the AIMS2 which asked patients to rate their overall health on a scale from 1 to 5, 1 being* excellent* and 5* poor*.

### 2.3. Statistical Analyses

Descriptive analyses were used to examine patient characteristics at baseline and variable distributions at 1 year. To test the impact of* pain intensity* at baseline on functional outcomes at 1 year, a series of logistic regression models were run separately for each patient-reported dichotomous measure of function as the dependent variable, with each measure of pain intensity as predictor in separate models.

To ensure that the impact on 1-year functional outcomes was not simply reflecting baseline functional limitations, baseline estimates of the corresponding functional measure were included in all models. The intervention of the parent study as well as sociodemographic factors commonly considered covariates in studies of pain and those significantly correlated with any of the outcome measures were considered for inclusion. Similarly, to ensure that overall health status at baseline did not explain associations, baseline measures of self-rated health were also explored for adjustment. To avoid spurious associations or loss of statistical power due to the addition of variables, fit of each model after inclusion of each variable identified as noted above was assessed using the likelihood ratio test. Only covariates that added significantly to the model were retained in subsequent models.

Parallel regression models were also run for pain measures other than pain intensity including the* AIMS2 pain composite score* (last month) and the* frequency of severe pain* in the last month as predictors. Once the initial pattern of outcomes was evaluated, to simplify interpretation, final analyses using a combined binary measure of function if any limitations were identified by both patient and physician report were estimated in similar fashion.

Finally, to clarify which items of the AIMS2 pain scale (i.e., pain intensity in the last month or frequency of severe pain in the last month) were driving the association with functional limitations, a stepwise backwards procedure was conducted with cut-off for inclusion of *p* < 0.05, predicting the physician and patient combined functional limitations one year later. For interpretability in clinical care, the single item of frequency of severe pain was also explored in its original categorical 5-point scale.

StataSE 11 [18] was used to conduct these analyses.

### 2.4. Human Subjects

The parent study was conducted in accordance with all human subjects' protections and IRB approvals. The present deidentified analyses were granted exemption by the University of Wisconsin IRB.

## 3. Results and Discussion

### 3.1. Results

A total of 262 subjects, representing 58% of the initial sample of the parent study, had complete data on all of the key variables in final analyses. Mean pain and functional outcomes were similar between the sample used and the full dataset (Table 1).

Pain was common among patients at baseline: 95% reported some level of pain in the last 24 hours or in the last month. Of the AIMS2 pain scale items,* pain in 2 or more joints* had the highest mean (5), followed by* frequency of severe pain* (4.2), while* pain that interferes with sleep* had the lowest mean (3.3). Correlations among measures of pain were moderate to high (Table 2). Pain intensities referencing the last month and last 24 hours were moderately correlated (*r*_*s*_ = 0.62). Of the two pain intensity measures, reference to the last month was more correlated with frequency of severe pain (also referencing the last month) (*r*_*s*_ = 0.76, *p* < 0.05) than pain intensity in the last 24 hours (*r*_*s*_ = 0.58, *p* < 0.05). Pain intensity in the last month and frequency of severe pain in the last month were even more highly correlated with the full AIMS2 pain composite score of which they are a part (*r*_*s*_ = 0.82 and 0.87, resp.).

Simple correlations among dichotomized measures of function were not as high (Table 2). At 1 year, agreement between dichotomized measures of mobility and self-care limitations was about 25% above chance (Kappa = 0.25 (*p* < 0.000)) and between either of these and the physician-rated ACR functional status dichotomized measure was 33% above chance agreement (Kappa = 0.33 (*p* < 0.000)). The measure that combines identified limitations by both patient and provider is intended to capture the potentially more extreme expressions of functional limitations. At 1 year, 42% of the sample was classified as having one or more functional limitations on both patient and physician report.

The 16 logistic regression models examining the association between each of the 4 pain measures and each of the 4 dichotomous outcome measures of functional limitations are summarized in Table 3. The intervention of the parent study was tested as a covariate but was not associated with pain or functional outcomes and did not add to the model. In similar fashion, systematic adjustments for baseline demographics and self-rated health did not add significantly to these models or change the main effects of interest and therefore, for statistical parsimony, were not included.

Each model represents the baseline pain measure estimating the given measure of functional limitation after adjustments for the corresponding baseline measure of function (Table 3). After these adjustments, pain intensity assessed using the last 24 hours as referenced at baseline significantly predicted 2 of the 3 specific functional outcomes examined, reaching statistical significance for ACR and self-care limitations. For every unit increase in baseline pain in the last 24 hours assessed at baseline, the odds of ACR limitations and self-care limitations at 1 year increased by 15% and 22%, respectively.

Conversely, pain intensity assessing the last month at baseline did not significantly predict self-care limitations but did significantly predict self-reported mobility limitations as well as physician ACR functional rating at 1 year. For a unit increase in pain intensity score assessed in the last month at baseline, the odds of ACR functional limitations increased by 23% and mobility by 15%, for 16% increased odds of functional limitations reported by both patient and physician.

**Figure 1:**
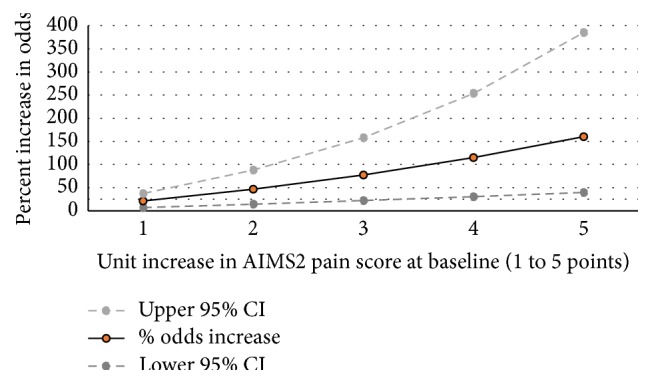
Percent increase in odds of functional limitations at 1 year associated with 1- to 5-point increases in baseline AIMS2 pain scores.

Models of AIMS2 composite pain scale predicting functional limitations at 1 year yielded the most consistent results—all models were statistically significant and of the greatest magnitude. Increased odds of functional limitations ranged between a high of 32% for self-care and 25% for ACR and 19% for mobility limitations. Overall, for every point increase in the AIMS2 pain scale at baseline, the odds of having functional limitations identified by both patient and physician were 21%. Results were similar, of slightly lower magnitude for estimates by the single item measuring frequency of severe pain in the last month (Table 3).  Figure 1 further characterizes results predicting functional limitations reported by both physician and patient, representing the percent increase in odds of functional limitations for 1- to 5-point increases in baseline AIMS2 pain scale score.

A stepwise backwards regression procedure including all individual items of the AIMS2 pain scale with the cut-off for inclusion of *p* < 0.05 confirmed that, other than baseline function, frequency of severe pain in the last month was the single item that remained in the model. Given the potential utility of this 1 item of the AIMS2 pain scale, to maximize interpretability for clinicians who may use the variable response options in their original form rather than in the manner scored to maximize comparability across pain measures, parallel models were run with the frequency of severe pain categorical variable using the lowest level of* no days* as referent category. Compared to those reporting* no days, *patients reporting* most days* had 3 times the odds of functional limitations and those reporting* all days* had 5 times increased odds of functional limitations reported by both patient and physician at 1 year (details not shown in table).

### 3.2. Discussion

Recommendations on measurement of clinical pain for clinical trials have identified the need for further research on the specifics of pain measurement for a range of purposes including the relevant time frames assessed and performance of single item versus composite measures of pain. However, studies seldom focus on how to best measure pain when researchers and clinicians are interested in how pain predicts future function. The failure of published studies to characterize details on the measurements of pain reduces interpretability of research findings and its implications for clinical practice. Even with the more widely studied concept of pain intensity, recent research underscores that nearly a quarter of reviewed publications did not provide adequate information on the measure of pain intensity used and a third failed to note time period assessed by the measure [19]. The present study has identified the AIMS2 pain scale (assessing arthritis pain in the last month) as more consistently predicting functional limitations reported by both patient and physician 1 year later and provides evidence and comparison of results obtained from different single item measures. Given the anticipated improved reliability of a multi-item measure versus a single item measure, this is not surprising.

Although all of the explored measures of pain predicted functional limitations in some domains, choice of measure does appear to matter for identifying statistically significant findings in the current sample. Pain intensity in the last 24 hours was the least likely to estimate a range of statistically significant functional limitations 1 year later based on the outcomes explored, whereas the composite AIMS2 pain scale, which references the last month, showed consistent results across measures of function. Based on the measure of function requiring both physician and patient identified limitations, while all other measures of pain identify a greater than 15% increase in function per unit increase in pain, pain intensity in the last 24 hours was not significant and the magnitude estimated was only a 4% increase in odds of functional limitations. Thus, based on data in our study, if we had used a single measure among these different pain measures, we may have reached very different conclusions.

Similarly, physicians have the opportunity to ask about current pain, pain in the last 24 hours or last month, or composite measures. Often, current pain intensity or in the last 24 hours is used. However, given these results, pain intensity in the last month may be more informative, although not for self-care limitations. Therefore, if a composite measure like the AIMS2 is not feasible, its constituent item, which asks about the frequency of severe pain in the last month, rather than pain intensity, may be nearly as useful as the composite measure in predicting statistically significant limitations identified across measures and by both physician and patient self-report one year later.

#### 3.2.1. Limitations

As in all research on pain, this study relied on self-reported pain which, although the most valid measure, may introduce error or bias. A primary limitation is that the measures of pain intensity assessing different time periods also differed slightly in wording of the original question, response options, and scale. The last 24 hours was assessed by a numerical rating scale with response options originally on a 0–10 scale, whereas pain in the last month was assessed using a verbal rating scale whose 5 categorical answer options were then transformed to a 0–10 scale as is done when combined with the remaining items that make up the AIMS2 pain scale. Despite this difference, both items measure pain intensity and were analyzed with a similar 0–10 metric for valid statistical comparison.

However, any measure expanding the time period referenced, that is, the last month rather than the last 24 hours, may introduce recall bias. This bias may work in two ways: (1) diluting the true effect by introducing random error or (2) introducing greater pain recall among those with current functional impairments. However, the pattern of results does not support either of these potential effects. The association with 1 year function was more consistent for pain in the last month than the last 24 hours, and this held even with adjustment of baseline function, making recall bias an unlikely explanation. Although measurement error may be underestimating all effects, there is no reason to assume that these measures are not capturing the two intended and related but distinct constructs of average pain over the last month versus a more acute pain experience over the last 24 hours.

The choice of functional outcomes is also a challenge in such research. For this reason we included self-reported functional outcomes in addition to clinician report using the ACR class rating. Each has limitations and thus including both helps provide a fuller picture. The ACR class rating provided more stable results which may not capture the same nuance as the self-reported measure of mobility and self-care limitations. For all measures of function, the choice of cut-off for categorizing functional limitations may have been too liberal and yielded a potentially heterogeneous group. Due to reductions in sample size with complete data needed for comparisons, the number of patients reporting limitations across the range of the original function scales was reduced, precluding valid use of the full quantitative scale, or categorization at a more extreme level consistent with potential clinical significance. For example, classes II, III, and IV on the ACR rating were combined as representing the presence of functional limitations. However, assuming that the association between pain and more extreme classification of functional limitations would be expected to be of greater magnitude, this potential limitation may have led to underestimating the magnitude of the association but does not invalidate these findings. Moreover, combining responses from both sources (patient and physician) provided a more stable and extreme group for which agreement on limitations was more likely.

Separately, although the authors draw inferences about the utility of pain measures to determine function one year later in clinical care, this study did not examine how pain was assessed in the clinical visit and how this information was used to change course of treatment. All measurements of pain were based on postvisit research instruments. This however does not invalidate the measurements of pain. Pain ratings were fairly well correlated with each other; pain was common in this sample and discussed during most visits. Based on these facts, it is reasonable to expect that the research-assessed pain measures correlated with ratings of pain during the clinical visit.

A final related potential limitation is that we do not adjust for RA disease activity. It is reasonable that disease activity would highly correlate with pain ratings. For example, is it actual disease activity, not simply pain, that is driving these results? It is reasonable to expect that higher pain at baseline would lead to more aggressive treatment, which would theoretically lead to better functional outcomes than if such treatment is not provided, suggesting that more aggressive treatment at baseline in response to high pain severity reports may have led us to underestimate the true relationship between pain severity and functional outcome one year later. Although we do not adjust for measures of RA disease activity, we did explore self-rated health at baseline as a proxy and although associated in some cases with function at 1 year, it did not explain these associations in models adjusted for baseline function.

## 4. Conclusions

These findings suggest that when making decisions relevant to improving long-term functional outcomes, physicians should definitely assess pain and the choice of measure and time frame may impact conclusions. When assessing pain, clinicians and researchers should consider composite measures such as the pain scale of the AIMS2. When resources and time allow, multiple items should be obtained. Given time constraints, clinicians may only have time for a single item. If single item* pain intensity* measures are used, focus on pain in the* past month* rather than just the* last 24 hours* may be prudent. However, when future function is of interest, measuring* frequency of severe pain* in the last month as the single item may be a more robust alternative.

## Figures and Tables

**Table 1:** Patient sociodemographic, pain, and functional characteristics at baseline and 1-year follow-up.

	Full sample		Sample for current analysis	
	Baseline	12 months	Baseline	12 months
Maximum *N*	*N* = 450	*N* = 351	*N* = 262	*N* = 262
% of full baseline sample	100	78	58	58
Sociodemographics				
Age (mean (SD))	62 (9.4)		61 (9.4)	
Female (%)	76		80	
White (%)	87		91	
Education (%)				
HS diploma or less	45		40	
Greater than HS	55		60	
Married (%)	67			
State (%)				
Wisconsin	56		76	
North Carolina	44		24	
Intervention arm (%)	51		52	
Pain levels				
Last 24 hours (0–10) (mean (SD))	3.8 (2.6)		3.6 (2.5)	
Last month (0–10) (mean (SD))^*∗*^	5.9 (2.6)		5.8 (2.6)	
Frequency of severe pain (0–10) (mean (SD))^*∗*^	4.2 (3.1)		4.2 (3.0)	
AIMS2 pain scale (0–10) (mean (SD))	4.5 (2.6)		4.4 (2.5)	
Morning stiffness	3.9 (3.5)		3.6 (3.5)	
Pain that interferes with sleep	3.3 (3.2)		3.3 (3.2)	
Pain in 2 or more joints	5.2 (3.2)		5.0 (3.1)	
Function levels				
ACR functional ≥ class II (%)	71	65	71	65
Mobility limitations ≥ 1 (%)	39	51	34	50
Self-care limitations ≥ 1 (%)	18	18	16	16
Self-rated health (1–5) mean (SD)	3.1 (1.0)		3.0 (1.0)	
Limitations identified by patient *and* physician (%)	35	42	30	42

^*∗*^Also AIMS2 pain scale items.

**Table 2:** Spearman correlations between variables of interest.

	(1)	(2)	(3)	(4)	(5)	(6)	(7)	(8)	(9)	(10)	(11)	(12)	(13)	(14)	(15)	(16)	(17)	(18)
Pain variables, baseline																		
(1) Pain in last 24 hrs	1																	
(2) Pain in last month	0.62^*∗*^	1																
(3) Frequency of severe pain	0.58^*∗*^	0.76^*∗*^	1															
(4) AIMS2 pain scale	0.66^*∗*^	0.82^*∗*^	0.87^*∗*^	1														
(5) AIMS2 pain scale, pain in 2+ joints	0.56^*∗*^	0.66^*∗*^	0.67^*∗*^	0.85^*∗*^	1													
(6) AIMS2 pain scale, morning stiffness	0.49^*∗*^	0.53^*∗*^	0.61^*∗*^	0.81^*∗*^	0.61^*∗*^	1												
(7) AIMS2 pain scale, sleep interference	0.49^*∗*^	0.55^*∗*^	0.62^*∗*^	0.79^*∗*^	0.56^*∗*^	0.56^*∗*^	1											
Functional limitation variables, baseline																		
(8) ACR functional classes II-III	0.27^*∗*^	0.32^*∗*^	0.33^*∗*^	0.37^*∗*^	0.33^*∗*^	0.29^*∗*^	0.26^*∗*^	1										
(9) Mobility limitations	0.28^*∗*^	0.29^*∗*^	0.33^*∗*^	0.34^*∗*^	0.29^*∗*^	0.30^*∗*^	0.23^*∗*^	0.12	1									
(10) Self-care limitations	0.22^*∗*^	0.25^*∗*^	0.33^*∗*^	0.32^*∗*^	0.22^*∗*^	0.24^*∗*^	0.28^*∗*^	0.14^*∗*^	0.40^*∗*^	1								
Functional limitation variables, 1 year																		
(11) ACR functional classes II-III	0.26^*∗*^	0.32^*∗*^	0.32^*∗*^	0.37^*∗*^	0.33^*∗*^	0.26^*∗*^	0.28^*∗*^	0.57^*∗*^	0.23^*∗*^	0.18^*∗*^	1							
(12) Mobility limitations	0.19^*∗*^	0.28^*∗*^	0.33^*∗*^	0.32^*∗*^	0.26^*∗*^	0.23^*∗*^	0.29^*∗*^	0.57^*∗*^	0.56^*∗*^	0.35^*∗*^	0.34^*∗*^	1						
(13) Self-care limitations	0.23^*∗*^	0.22^*∗*^	0.33^*∗*^	0.30^*∗*^	0.21^*∗*^	0.25^*∗*^	0.27^*∗*^	0.57^*∗*^	0.30^*∗*^	0.56^*∗*^	0.24^*∗*^	0.34^*∗*^	1					
Sociodemographic variable, baseline																		
(14) Age	−0.04	0.12	0.07	−0.01	−0.06	−0.11	−0.03	0.12^*∗*^	0.11	0.04	0.09	0.13^*∗*^	0.12	1				
(15) Greater than high school	−0.09	−0.19^*∗*^	−0.23^*∗*^	−0.21^*∗*^	−0.11	−0.20^*∗*^	−0.14^*∗*^	−0.02	−0.15^*∗*^	−0.09	−0.04	−0.19^*∗*^	−0.13^*∗*^	−0.19^*∗*^	1			
(16) Self-rated health	0.42^*∗*^	0.41^*∗*^	0.47^*∗*^	0.55^*∗*^	0.46^*∗*^	0.47^*∗*^	0.47^*∗*^	0.26^*∗*^	0.39^*∗*^	0.33^*∗*^	0.25^*∗*^	0.36^*∗*^	0.28^*∗*^	0.01	−0.16^*∗*^	1		
(17) State	−0.20^*∗*^	−0.13^*∗*^	−0.17^*∗*^	−0.16^*∗*^	−0.11	−0.11	−0.15^*∗*^	−0.07	−0.24^*∗*^	−0.25^*∗*^	−0.03	−0.20^*∗*^	−0.23^*∗*^	0.03	0.07	−0.14^*∗*^	1	
(18) Married	−0.10	−0.13^*∗*^	−0.09	−0.05	0.01	0.01	−0.02	0.04	−0.17^*∗*^	0.07	0.04	−0.15^*∗*^	0.04	−0.08	0.04	0.03	0.18^*∗*^	1
(19) White	−0.15^*∗*^	−0.07	−0.13^*∗*^	−0.16^*∗*^	0.13^*∗*^	0.15^*∗*^	0.17^*∗*^	0.00	−0.26^*∗*^	−0.20^*∗*^	−0.09	−0.21^*∗*^	−0.20^*∗*^	−0.01	0.05	−0.13^*∗*^	0.47^*∗*^	0.14^*∗*^

^*∗*^ *p* values < 0.05; *N* = 254. *Intervention arm versus control* and *gender* were omitted because no correlations were appreciable or statistically significant.

**Table 3:** Logistic regression models of baseline pain ratings predicting functional limitations at 1 year.

Predictors (baseline)		Outcomes: functional limitations (1 year)										
		Physician report of function			Patient report of function						Physician and patient	
		ACR classes II–IV			Mobility			Self-care			Report of function	
		OR	95% CI		OR	95% CI		OR	95% CI		OR	95% CI
Models for pain in the last 24 hours:												
Pain in last 24 hours		**1.15**	(1.01, 1.31)		1.05	(0.93, 1.220)		**1.22**	(1.03, 1.43)		1.04	(0.92, 1.18)
Corresponding functional limitations		13.22	(6.84, 25.54)		15.45	(7.48, 31.90)		15.72	(6.95, 35.56)		13.17	(6.57, 26.38)

Models for pain in the last month:												
Pain in the last month		**1.23**	(1.08, 1.40)		**1.15**	(1.02, 1.29)		1.18	(0.99, 1.40)		**1.16**	(1.03, 1.32)
Corresponding functional limitations		11.96	(6.14, 23.29)		14.54	(7.09, 29.81)		15.54	(6.86, 35.19)		10.99	(5.50, 21.95)

Models for full AIMS2 pain scale:												
AIMS2 pain scale		**1.25**	(1.10, 1.43)		**1.19**	(1.05, 1.34)		**1.32**	(1.11, 1.59)		**1.21**	(1.07, 1.37)
Corresponding functional limitations		11.28	(5.77, 22.07)		13.56	(6.58, 27.92)		12.9	(5.60, 29.71)		10.32	(5.16, 20.65)

Models for frequency of severe pain:												
Frequency of severe pain		**1.18**	(1.06, 1.33)		**1.16**	(1.05, 1.29)		**1.25**	(1.09, 1.45)		**1.19**	(1.07, 1.32)
Corresponding functional limitations		12.04	(6.20, 23.40)		13.72	(6.67, 28.23)		12.56	(5.45, 28.95)		10.54	(5.29, 21.00)

*N* = 262; OR = odds ratio; CI = confidence interval.

Bold odds ratios represent significant main effects of baseline pain predicting function at 1 year at *p* < 0.05.
